# Bisphosphonates and risk of cancers: a systematic review and meta-analysis

**DOI:** 10.1038/s41416-020-01043-9

**Published:** 2020-09-09

**Authors:** Yuan-Yuan Li, Li-Jie Gao, Yu-Xue Zhang, Shu-Juan Liu, Shuo Cheng, Yu-Peng Liu, Cun-Xian Jia

**Affiliations:** 1 Department of Epidemiology, School of Public Health, Cheeloo College of Medicine, Shandong University, Jinan, China; 2 Department of Preventive Medicine, Public Health School, Harbin Medical University, Harbin, China; 3 Department of Preventive Medicine, School of Public Health and Management, Wenzhou Medical University, Wenzhou, China

**Keywords:** Cancer prevention, Cancer prevention

## Abstract

**Background:**

It is unclear whether bisphosphonates are associated with risk of cancers. Therefore, this meta-analysis aimed to evaluate the effect of bisphosphonates on overall cancers.

**Methods:**

A search in Pubmed, Embase, Cochrane Library and Web of Science databases was conducted, from the inception date of each resource to September 26, 2019. The summarised effect estimates with 95% CIs were calculated using a random-effect model. Heterogeneity and publication bias were explored.

**Results:**

Thirty-four articles were included in this study (4,508,261 participants; 403,196 cases). The results revealed that bisphosphonates significantly decreased the risk of colorectal cancer (RR = 0.89, 95% CI: 0.81–0.98), breast cancer (RR = 0.87, 95% CI: 0.82–0.93) and endometrial cancer (RR = 0.75, 95% CI: 0.61–0.94), but no significant association was observed in all-cause cancer. Furthermore, nitrogen-containing bisphosphonates only had protective effects both on breast cancer (RR = 0.94, 95% CI: 0.90–0.99) and endometrial cancer (RR = 0.70, 95% CI: 0.54–0.92). Non-nitrogen-containing bisphosphonates tended to increase the risk of liver cancer (RR = 2.14, 95% CI: 1.23–3.72) and pancreas cancer (RR = 1.75, 95% CI: 1.32–2.33).

**Conclusion:**

Bisphosphonates are significantly associated with risk reduction of colorectal, breast and endometrial cancer, especially nitrogen-containing bisphosphonates. It should be noted that non-nitrogen-containing bisphosphonates might increase the risk of liver and pancreas cancer. Large prospective cohort studies are needed to find the causal association between bisphosphonates and risk of cancers.

## Background

Cancer is an important public health problem in the world, and the incidence and mortality of global cancer have grown rapidly in recent years.^1^ According to the Global Burden of Disease study (GBD) 2017, the most common incident cancers were non-melanoma skin cancer (NMSC, 7.7 million incident cases), tracheal, bronchus and lung (TBL) cancer (2.2 million incident cases), breast cancer (2.0 million incident cases) and colorectal cancer (1.8 million incident cases).^2^ In terms of women, breast cancer was the most commonly diagnosed cancer, followed by colorectal, lung and cervical cancer.^3^ Prevention of cancers has become a great public health importance.

In recent years, preclinical studies have suggested that bisphosphonates have direct and indirect antitumour properties, including inhibition of tumour cell adhesion and proliferation,^4,5^ induction apoptosis of tumour cells,^6^ prevention of angiogenesis^7,8^ as well as activation of immune cells.^9^ Wysowski et al.^10^ reported that the US Food and Drug Administration (FDA) received 23 cases of oesophageal cancer after the use of bisphosphonates. Since then, some epidemiological studies were also conducted on the association between bisphosphonates and the risk of some types of cancers, but the results of these studies were controversial.^11–14^ To date, most meta-analyses were focused on the association between the use of bisphosphonates and the risk of some specific types of cancers.^15–24^ For instance, Yang et al.^19^ suggested that the use of bisphosphonates might decrease the risk of colorectal cancer by 11% (RR = 0.89, 95% CI: 0.80–0.99), while Oh et al.^25^ found no significant association between the use of bisphosphonates and colorectal cancer (RR = 0.62, 95% CI: 0.30–1.29). To the best of our knowledge, there was currently only one meta-analysis by Deng et al.^26^ on the use of bisphosphonates and the risk of all-cause cancers, which only included 13 cohort studies and analysed the association in mixed genders and females.

Bisphosphonates are widely prescribed for preventing and treating osteoporosis,^27,28^ but the number of bisphosphonate users is expected to increase globally. For example, in the United States alone, there are approximately 40 million bisphosphonate prescriptions each year. Considering the widespread use of bisphosphonates, it is essential to explore the association between bisphosphonates and cancers.

This systematic review and meta-analysis was intended to (1) analyse possible association between the use of bisphosphonates and the risk of overall cancers and individual types of cancers based on observational studies, (2) stratify analysis by different types and duration of bisphosphonates.

## Methods

### Literature search

This systematic review and meta-analysis was conducted following the PRISMA guidelines.^29^ We firstly searched relevant studies in the databases of Pubmed, Embase, Cochrane Library and Web of Science from the inception date of each resource to September 26, 2019, by two study investigators (Li and Cheng) independently. Detailed search terms were shown in Supplementary Table S1. Then, before the statistical analysis of the data, we manually searched from lists of references cited by the published studies, or updated our studies from other sources upto December 7, 2019, to identify whether there was new literature published (PROSPERO registration number is CRD4-2014014901).

### Selection criteria

The selection criteria of this meta-analysis were composed of inclusion and exclusion criteria. Studies were included in this meta-analysis if they complied with the following criteria:

1. Study design was observational study (cohort study, case–control study, nested case–control study or case cohort study) addressing the association between the use of bisphosphonates and risk of any type of cancers
2. The exposure was defined as one or more prescriptions of bisphosphonates
3. The outcome was the incidence of cancers
4. Studies reported effect estimates, including odds ratios (ORs), relative risks (RRs) or hazard ratios (HRs) with 95% confidence intervals (CIs), or provided sufficient data to calculate them
5. If there were duplicate articles, the most recent published or the most complete data would be included
6. Language was restricted to English.

Accordingly, these studies were excluded:

1. Cross-sectional studies, reviews, comments or conference abstracts
2. Studies were in vitro or animal experiments
3. Studies without enough data to calculate the effect estimates

### Data extraction and quality assessment

Data extraction and quality assessment were independently implemented by two researchers (Li and Liu) by using standardised forms, and any disagreement was resolved through discussion until consensus was reached. The following information was extracted from each study: first author, year of publication, population location, study design, study period, sample size, the number of cases, participants’ age and sex, type and duration of bisphosphonates, type of cancers, adjusted confounding factors and the available effect estimates with the corresponding 95% CIs. The original authors of studies would be contacted, if the required information was missing.

The quality of each included study was assessed using the Newcastle–Ottawa Scale (NOS).^30^ The NOS assessed quality from the following three aspects: selection, comparability and exposure (case–control studies) or outcome (cohort studies). The total score of NOS was nine stars. Studies with a score of more than 6 stars were considered as relatively high quality. Conversely, studies with a score of less than 6 stars were considered as relatively low quality.

### Data synthesis and analysis

RRs were often used as indicators to assess the association between the use of bisphosphonates and risk of cancers, and HRs were similar to RRs. We pooled the risk estimates of case–control and cohort studies in the primary meta-analysis because ORs and RRs could provide similar risk estimates when the outcomes were rare.^31^ The maximally adjusted risk estimates with 95% CIs were pooled by using random-effect models to obtain a more conservative outcome. Then, the heterogeneity among studies was evaluated by using the Cochrane’s *Q* statistic and the *I*^2^ statistic.^32^ The low, moderate and high degrees of heterogeneity in this study corresponding to that of the *I*^2^ cut-offs were 25%, 50% and 75%, respectively.

To explore the sources of heterogeneity among studies, random-effect meta-regression analysis based on the residual maximum likelihood (REML) method and subgroup analysis for all-cause cancers were conducted by study design, population region and sample size. According to the different molecular modes, bisphosphonates were classified into two groups, including nitrogen-containing bisphosphonates (such as alendronate, ibandronate, pamidronate, risedronate and zoledronate) and non-nitrogen-containing bisphosphonates (such as clodronate and etidronate).^33^ Then, the subgroup analysis was carried out to study the association between the use of bisphosphonates and various types of cancers based on different types (nitrogen- and non-nitrogen-containing bisphosphonates) and duration (<1 year and ≥1 year) of bisphosphonates.

The sensitivity analysis was performed by omitting one study and calculating the pooled risk estimates with 95% CIs of the remaining studies. Furthermore, the potential publication bias was evaluated with funnel plots,^34^ and was quantitatively examined by Egger’s linear regression tests.^35^ If there was publication bias, we would adjust the effect by using the trim-and-fill method.^36^ For statistical tests, a two-sided *P* < 0.05 was considered statistically significant. All statistical analyses were performed using STATA version 15.1 (Stata Corporation, College Station, TX, USA).

## Results

### Literature search and study characteristics

**Fig. 1:**
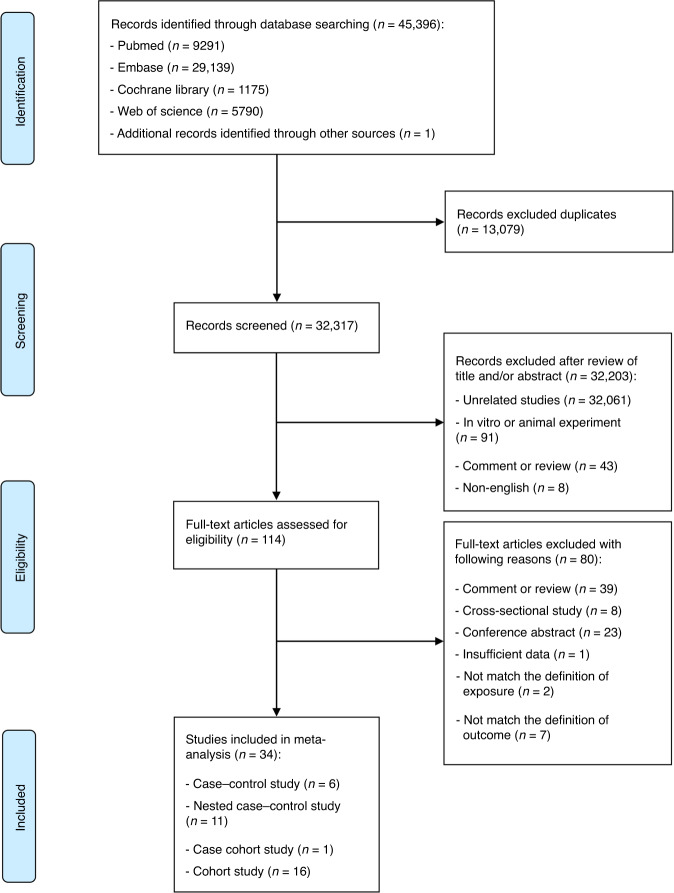
Flow diagram of study selection in this meta-analysis. The update search was carried out on December 7, 2019, also manually searched lists of references cited by the published studies to examine for any additional studies reporting primary data.

The flow diagram describing detailed literature searches and selection process is shown in Fig. 1. In brief, we identified 45,396 articles through systemically searching in the databases, of which 34 eligible articles (16 cohort studies,^11,13,14,37–49^ 11 nested case–control studies,^12,50–59^ 6 case–control studies^60–65^ and 1 case cohort study^66^) were included in the current meta-analysis.

The main characteristics of the included studies are summarised in Table 1. In total, 14 researches originated from Europe, 12 from America and 8 from Asia. Most of the papers (21/34) were published from 2010 to 2013. Of these, the data were available from 4,508,261 participants, including 403,196 cases originating from 19 types of cancers. Some articles focused on multiple types of cancers: 24 papers reported gastrointestinal cancers (stomach, small bowel, colon and rectum), 16 papers reported gynaecological cancers (endometrium, cervix and ovary), 14 papers reported oesophageal cancer, 10 papers reported breast cancer, 7 papers reported hepatobiliary cancers (liver and bladder), 5 papers reported lung cancer, 3 papers reported prostate cancer and 14 papers reported other cancers. Most of the participants were women, and the mean age ranged from 54.5 to 74.3 years. These studies indicated that alendronate was one of the most commonly used bisphosphonates. In addition, all studies adjusted confounding factors, and the quality assessment of the overall studies was high. Details of the adjusted confounding factors and quality assessment can be seen in Supplementary Tables S2–S4.

**Table 1:** The main characteristics of 34 included studies.

Study [year]	Population location	Study design	Study period	Sample size	Number of cases	Mean age (years)^a^	Sex, female (%)	Types of bisphosphonates	Duration of bisphosphonates	Types of cancers
Fortuny et al. ^63^	USA	Case–control study	2001–2005	936	469	63.6 ± NA	100	NA	NA	Endometrial cancer
Newcomb et al.^61^	USA	Case–control study	2003–2006	5911	2936	54.5 ± 8.9	100	NA	3–12 months; 13–24 months; ≥25 months	Breast cancer
Rennert et al.^65^	Northern Israel	Case–control study	2000–2010	4039	1832	64.7 ± NA	100	Alendronate: 86.7%	<1 year; 1–2 years; 2–3 years; 3–4 years; 4–5 years; >5 years	Breast cancer
Rennert et al.^64^	Northern Israel	Case–control study	2000–2006	1866	933	71.6 ± NA	100	Alendronate: 94.7%	<1 year; 1–2 years; 2–3 years; >3 years	Colorectal cancer
Wright et al. ^60^	UK	Case–control study	1995–2007	43,180	8636	64.7 ± NA	36.4	NA	NA	Oesophageal and gastric cancer
Rennert et al.^62^	Israel	Case–control study	2003–2010	805	464	67.2 ± NA	100	Alendronate and risedronate	<1 year; 1–2 years; 2–3 years; 3–4 years; 4–5 years; >5 years	Endometrial and ovarian cancer
Green et al.^59^	UK	Nested case–control study	NA	93,678	15,613	≥40	NA	NA	NA	Oesophageal, gastric and colorectal cancer
Nguyen et al.^56^	USA	Nested case–control study	2000–2002	812	116	64.7 ± 10.3	2.6	Alendronate, risedronate, etidronate, tiludronate and ibandronate	≥1 prescription	Oesophageal cancer
Chen et al. ^50^	Taiwan, China	Nested case–control study	2001–2008	3093	282	NA	NA	Alendronate	<1 year; ≥1 year	Oesophageal cancer
Singh et al. ^52^	Canada	Nested case–control study	2000–2009	59,667	5425	≥50	46.0	Alendronate, risedronate, etidronate, pamidronate, clodronate and zoledronate	<210 days; 210–572 days; 573–1250 days; >1250 days	Colorectal cancer
Vinogradova et al.^53^	UK	Nested case–control study	1997–2011	1,023,458	180,401	69.5 ± 10.0	49.7	Alendronate, risedronate, etidronate and ibandronate	<1 year; ≥1 year	Non-gastrointestinal cancers^b^
Vinogradova et al.^54^	UK	Nested case–control study	1997–2011	217,569	28,625	≥50	49.7	Alendronate, etidronate, ibandronate and risedronate	<1 year; ≥1 year	Oesophageal, gastric and colorectal cancer
Vogtmann et al.^55^	California	Nested case–control study	1997–2011	146,567	2934	67.2 ± 12.1	30.8	NA	<1 year; ≥1 year	Oesophageal and gastric cancer
Jung et al. ^12^	Korea	Nested case–control study	2002–2013	8540	1708	≥40	66.6	Alendronate, risedronate, etidronate, ibandronate, clodronate and pamidronate	NA	Oesophageal and gastric cancer
Busby et al.^57^	Scotland	Nested case–control study	1993–2011	18,035	3098	69.2 ± 11.2	35.4	NA	NA	Oesophageal and gastric cancer
Vogtmann et al.^58^	California	Nested case–control study	1997–2011	612,039	12,505	65.9 ± 12.9	48.5	Alendronate, risedronate, etidronate, ibandronate and tiludronate	<1 year; ≥1 year	Colorectal cancer
Chung et al.^51^	Denmark	Nested case–control study	1996–2013	30,228	2748	NA	37.4	Alendronate, risedronate, etidronate, ibandronate, clodronate and zoledronate	≤3 months; 3–12 months; >12 months	Renal cell carcinoma
Chiang et al.^66^	Taiwan, China	Case cohort study	1998–2009	27,603	3467	73.5 ± 8.4	100	Alendronate	≤2 years; >2 years	All types of cancer
Abrahamsen et al.^40^	Denmark	Cohort study	1995–2005	41,034	85	74.3 ± 8.8	89.1	Alendronate, risedronate, etidronate, ibandronate and clodronate	Mean 2.1 years	Oesophageal and gastric cancer
Cardwell et al.^38^	UK	Cohort study	1996–2006	83,652	314	70.0 ± 11.4	81.0	Alendronate, risedronate, etidronate, ibandronate, tiludronate and clodronate	NA	Oesophageal cancer
Chlebowski et al.^41^	USA	Cohort study	1993–2005	154,768	6276	50–79	100	Alendronate, etidronate, tiludronate and pamidronate	<1 year; 1–3 years; ≥3 years	Breast cancer
Vestergaard et al.^11^	Denmark	Cohort study	1996–2006	414,245	103,562	70.5 ± 11.4	84.7	Alendronate, etidronate and clodronate	NA	Digestive system cancers^c^
Vestergaard et al.^48^	Denmark	Cohort study	1996–2006	348,426	884	71.1 ± 10.7	100	Alendronate, risedronate, etidronate, ibandronate, clodronate, pamidronate and zoledronate	NA	Breast cancer
Abrahamsen et al.^37^	Denmark	Cohort study	1996–2005	153,030	318	71.9 ± 10.0	100	Alendronate	NA	Oesophageal and gastric cancer
Cardwell et al.^13^	UK	Cohort study	1996–2006	83,652	5956	70.0 ± 11.4	81.0	NA	< 1 year; 1–2 years; 2–3 years; 3–4 years	All types of cancer
Khalili et al.^45^	USA	Cohort study	1998–2008	86,277	801	64.7 ± 7.1	100	Alendronate, risedronate, etidronate and other bisphosphonates	1–2 years; 3–4 years; ≥5 years	Colorectal cancer
Lee et al.^39^	Taiwan, China	Cohort study	1998–2009	21,918	873	NA	84.4	Alendronate	NA	All types of cancer
Pazianas et al.^47^	Denmark	Cohort study	1996–2005	153,030	1683	71.9 ± 10.0	100	Alendronate	NA	Colon cancer
Passarelli et al.^14^	USA	Cohort study	1993–2009	143,335	1931	63.2 ± 7.2	100	Alendronate, risedronate, etidronate and tiludronate	<1 year; 1–3 years; ≥3 years	Colorectal cancer
Alford et al.^46^	USA	Cohort study	1993–2001	23,485	97	55–74	100	Alendronate, risedronate, etidronate and ibandronate	NA	Endometrial cancer
Newcomb et al.^43^	USA	Cohort study	1993–2010	83,286	1123	63.0 ± 7.2	100	Alendronate, risedronate, etidronate and tiludronate	<1 year; 1–3 years; ≥3 years	Endometrial cancer
Fournier et al.^49^	France	Cohort study	2004–2011	64,438	2407	62.8 ± 6.4	100	Alendronate, risedronate, etidronate, ibandronate, tiludronate and zoledronate	<0.5 year; 0.5–1 years; 1–3 years; ≥3 years	Breast cancer
Tao et al.^42^	USA	Cohort study	1993–2013	151,134	2511	50–79	100	Alendronate, risedronate, etidronate, tiludronate, pamidronate and zoledronate	<0.67 years; 0.67–1.49 years; 1.50–2.99 years; ≥3 years	Lung cancer
Bae et al.^44^	Korea	Cohort study	2003–2013	204,525	2183	57.2 ± NA	100	Alendronate and risedronate	NA	Female cancer^d^

*NA*  not available.

^a^If mean values of age were unavailable, median or range was extracted.

^b^Non-gastrointestinal cancers: bladder, breast, cervical, endometrial, lung, melanoma, ovarian, pancreas and prostate cancer.

^c^Digestive system cancers: oesophageal, liver, pancreas, colon and small intestinal cancer.

^d^Breast, endometrial and ovarian cancer.

### Bisphosphonates and risk of cancers

**Fig. 2:**
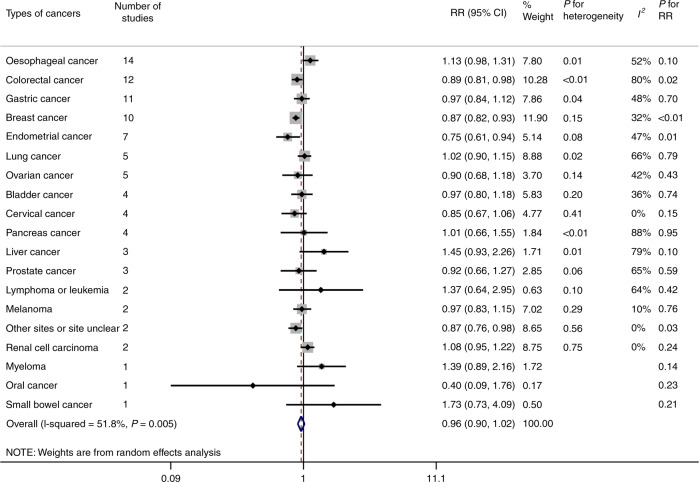
Forest plot of summary risk estimates in different types of cancers. The forest plot shows the RRs of different types of cancers comparing individuals using bisphosphonates to those without bisphosphonates. The types of cancers and the number of corresponding studies are shown in the figure. Each small rhombuses represents the RR for each type of cancer, with the location of the rhombuses representing both the direction and magnitude of the effect size and the horizontal line representing their 95% CIs. The square represents the weight of studies of each type of cancer. The hollow rhombus represents the pooled RRs. The maximally adjusted risk estimates with 95% CIs were pooled by using random-effect models to obtain a more conservative outcome. The heterogeneity among studies was evaluated by using the Cochraneʼs *Q* statistic and the *I*^*2*^ statistic, corresponding to the values of *P* for heterogeneity and *I*^*2*^ in the figure. *RR* risk ratio; *CI* confidence interval.

Fig. 2 shows the association between the use of bisphosphonates and risk of different types of cancers. Based on the estimates of the random-effect model, for specific types of cancers, the use of bisphosphonates was strongly associated with colorectal cancer (pooled RR = 0.89, 95% CI: 0.81–0.98, *P* = 0.02), breast cancer (pooled RR = 0.87, 95% CI: 0.82–0.93, *P* < 0.01) and endometrial cancer (pooled RR = 0.75, 95% CI: 0.61–0.94, *P* = 0.01). There was no statistically significant association between the use of bisphosphonates and oesophageal cancer (pooled RR = 1.13, 95% CI: 0.98–1.31, *P* = 0.10) as well as other types of cancers. The heterogeneity between studies was high in colorectal cancer (*P* < 0.01, *I*^*2*^ = 80%), pancreas cancer (*P* < 0.01, *I*^*2*^ = 88%) and liver cancer (*P* = 0.01, *I*^*2*^ = 79%), while the heterogeneity of the remaining studies was moderate and low. We found that the association between the use of bisphosphonates and risk of all cancers was not statistically significant (pooled RR = 0.96, 95% CI: 0.90–1.02, *P* = 0.18), with moderate heterogeneity between studies (*P* < 0.01, *I*^*2*^ = 52%). Detailed meta-analyses for each type of cancers are in Supplementary Fig. S1.

### Meta-regression and subgroup analysis

Random-effect meta-regression model and subgroup analysis were used to explore the primary heterogeneity in regard to population region, study design and sample size. The association between the use of bisphosphonates and risk of all cancers was weaker in the group of sample size ≥ 5000 than in the group of sample size < 5000 (*P* < 0.01). We found no evidence that population region (Asia: *P* = 0.88; Europe: *P* = 0.22) and study design (*P* = 0.65) influenced the association between the use of bisphosphonates and risk of all cancers (Table 2).

**Table 2:** Meta-regression and subgroup analyses with respect to all cancers.

Subgroups	Number of studies	RR (95%CI)	*I*^2^	P*
Overall	34	0.96 (0.90–1.02)	52%	
Population region				
America	14	**0.88 (0.82**–**0.95)**	38%	Ref.
Asia	8	0.88 (0.74–1.03)	75%	0.88
Europe	12	0.96 (0.89–1.03)	88%	0.22
Study design				
Case–control	18	0.95 (0.89–1.01)	70%	Ref.
Cohort	16	**0.92 (0.88**–**0.97)**	79%	0.65
Sample size				
<5000	6	**0.71 (0.62**–**0.83)**	0%	Ref.
≥5000	28	**0.94 (0.90**–**0.99)**	81%	<0.01

*P values were estimated by meta-regression.

The bold values represent *P* value < 0.05.

The subgroup analysis was conducted based on different types of bisphosphonates for various types of cancers (Table 3). Nitrogen-containing bisphosphonates had a protective effect on breast cancer (RR = 0.94, 95% CI: 0.90–0.99, *I*^*2*^ = 0%) and endometrial cancer (RR = 0.70, 95% CI: 0.54–0.92, *I*^*2*^ = 33%), but this effect was only observed in non-nitrogen-containing bisphosphonates on breast cancer (RR = 0.88, 95% CI: 0.81–0.95, *I*^*2*^ = 39%). Notably, non-nitrogen-containing bisphosphonates tended to increase the risk of liver cancer (RR = 2.14, 95% CI: 1.23–3.72) and pancreas cancer (RR = 1.75, 95% CI: 1.32–2.33).

**Table 3:** Subgroup analyses with respect to types of bisphosphonates.

Subgroups^a^	Nitrogen-containing bisphosphonates				Non-nitrogen-containing bisphosphonates			
	Number of studies	RR (95% CI)	*I*^2^	*P* for heterogeneity	Number of studies	RR (95% CI)	*I*^2^	*P* for heterogeneity
Oesophageal cancer	8	1.10 (0.88–1.38)	53%	0.04	3	1.36 (0.97–1.90)	57%	0.10
Gastric cancer	6	0.91 (0.75–1.10)	30%	0.21	2	1.07 (0.53–2.17)	87%	<0.01
Small-bowel cancer	1	2.19 (0.46–10.41)	NA	NA	1	1.56 (0.56–4.36)	NA	NA
Colorectal cancer	7	0.93 (0.79–1.09)	87%	<0.01	4	0.95 (0.85–1.07)	35%	0.20
Liver cancer	3	1.36 (0.90–2.04)	65%	0.06	1	**2.14 (1.23**–**3.72)**	NA	NA
Bladder cancer	2	1.18 (0.60–2.29)	47%	0.17	1	1.41 (0.79–2.53)	NA	NA
Pancreas cancer	2	1.11 (0.77–1.62)	7%	0.30	1	**1.75 (1.32**–**2.33)**	NA	NA
Renal cell carcinoma	2	1.15 (0.77–1.72)	0%	0.99	1	1.18 (0.94–1.49)	NA	NA
Breast cancer	6	**0.94** (**0.90**–**0.99)**	0%	0.68	2	**0.88 (0.81**–**0.95)**	39%	0.20
Cervical cancer	3	0.75 (0.55–1.01)	0%	0.47	NA	NA	NA	NA
Endometrial cancer	5	**0.70** (**0.54**–**0.92)**	33%	0.20	1	0.40 (0.06–2.74)	NA	NA
Ovarian cancer	3	0.89 (0.47–1.69)	47%	0.15	NA	NA	NA	NA
Prostate cancer	2	1.16 (0.56–2.39)	78%	0.03	1	0.98 (0.84–1.14)	NA	NA
Lung cancer	4	1.05 (0.91–1.22)	70%	0.02	2	1.40 (0.77–2.54)	80%	0.02

*NA*  not available.

^a^Lymphoma or leukaemia and oral cancer were not listed in the table because the number of studies was too few to analyse.

The bold values represent *P* value < 0.05.

Regarding the association between the duration of bisphosphonates and cancers, the use of bisphosphonates upto at least 1 year (RR = 0.78, 95% CI: 0.63–0.98, *I*^*2*^ = 92%) had a greater protective effect on breast cancer than their use of less than 1 year (RR = 0.90, 95% CI: 0.84–0.97, *I*^*2*^ = 0%). Moreover, we observed a significant risk reduction for the use of bisphosphonates upto at least 1 year on prostate cancer (RR = 0.85, 95% CI: 0.76–0.95). However, there was no significant association on other types of cancers (Table 4).

**Table 4:** Subgroup analyses with respect to duration of bisphosphonates.

Subgroups^a^	<1 year				≥1 year			
	Number of studies	RR (95% CI)	*I*^2^	*P* for heterogeneity	Number of studies	RR (95% CI)	*I*^2^	*P* for heterogeneity
Oesophageal cancer	5	1.22 (0.83–1.78)	66%	0.02	5	0.99 (0.79–1.24)	26%	0.25
Gastric cancer	3	1.11 (0.91–1.34)	0%	0.56	3	1.15 (0.75–1.75)	76%	0.02
Colorectal cancer	5	1.04 (0.88–1.24)	65%	0.02	7	0.84 (0.71–1.04)	89%	<0.01
Bladder cancer	2	1.59 (0.53–4.75)	74%	0.05	2	0.98 (0.64–1.48)	23%	0.25
Pancreas cancer	2	1.25 (0.44–3.52)	89%	<0.01	2	0.99 (0.58–1.68)	65%	0.09
Renal cell carcinoma	1	1.06 (0.73–1.53)	NA	NA	1	1.10 (0.78–1.56)	NA	NA
Breast cancer	4	**0.90** (**0.84**–**0.97)**	0%	0.88	5	**0.78** (**0.63**–**0.98)**	92%	<0.01
Endometrial cancer	2	0.89 (0.61–1.30)	0%	0.34	2	0.55 (0.27–1.12)	94%	<0.01
Ovarian cancer	2	0.75 (0.21–2.73)	43%	0.18	2	0.73 (0.41–1.30)	90%	<0.01
Prostate cancer	1	0.90 (0.79–1.02)	NA	NA	1	**0.85** (**0.76**–**0.95)**	NA	NA
Lung cancer	1	1.10 (1.00–1.21)	NA	NA	1	1.01 (0.92–1.10)	NA	NA
Melanoma	1	0.90 (0.73–1.10)	NA	NA	1	1.09 (0.92–1.29)	NA	NA

*NA* not available.

^a^Other cancers (small-bowel, liver, cervical cancer, lymphoma or leukaemia and oral cancer) were not listed in the table because the number of studies was too few to analyse.

The bold values represent *P* value < 0.05.

### Sensitivity analysis

We performed a sensitivity analysis by removing one study at each turn and calculating the pooled risk estimates with 95% CIs of the remaining studies. Two studies that were conducted by Abrahamsen et al.^37,40^ had an impact on the results in oesophageal cancer (pooled RR = 1.13, 95% CI: 0.98–1.31; removed Abrahamsen et al. 2012: RR = 1.17, 95% CI: 1.01–1.35; removed Abrahamsen et al. 2009: RR = 1.17, 95% CI: 1.03–1.33). Besides, the association was not materially changed in this analysis in other types of cancers (Supplementary Figs. S2–S5).

### Publication bias

**Fig. 3:**
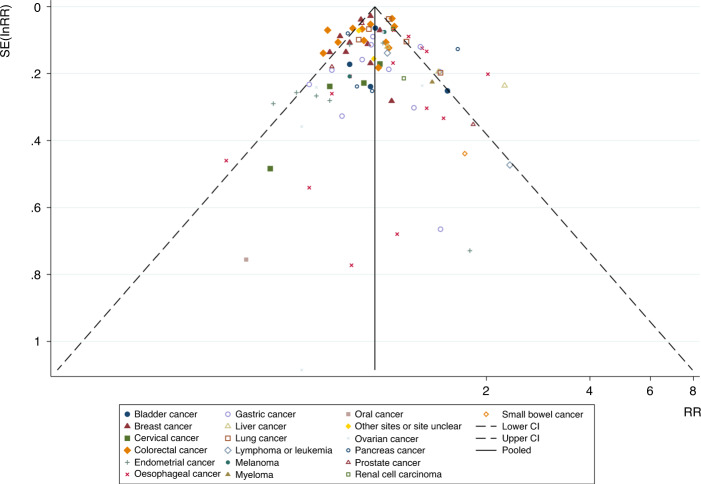
Funnel plot of the meta-analysis of the association between bisphosphonates and risk of all cancers. The abscissa is the effect size (RR), and the ordinate is the standard error of the effect size (SE (InRR)). Small symbols of different shapes represent the studies involved in different cancers. The vertical line in the middle represents the combined RR value, and the two oblique dashed lines represent the 95% confidence intervals of the funnel graph. The funnel plot of the association between the use of bisphosphonates and the risk of overall cancers as well as each type of cancer did not indicate substantial asymmetry.

The funnel plot of the association between the use of bisphosphonates and the risk of overall cancers as well as each type of cancer did not indicate substantial asymmetry (Fig. 3). For a specific type of cancers (including more than 10 articles), the Egger’s liner regression test also implied no evidence of publication bias (all cancers: Egger’s *P* = 0.83; oesophageal cancer: Egger’s *P* = 0.45; colorectal cancer: Egger’s *P* = 0.13; gastric cancer: Egger’s *P* = 0.92; breast cancer: Egger’s *P* = 0.26) (Table 5). Potential publication bias was not found through the funnel plot and the Egger’s liner regression test, so we did not carry out the trim-and-fill method.

**Table 5:** The bias examination of the association between bisphosphonates and risk of cancers.

Types of cancers	Number of studies	*P* value for Egger’s liner regression test
All cancers	34	0.83
Oesophageal cancer	14	0.45
Colorectal cancer	12	0.13
Gastric cancer	11	0.92
Breast cancer	10	0.26

## Discussion

### Summary of the main results

The major findings of this meta-analysis are (1) the use of bisphosphonates might have a protective effect on colorectal, breast and endometrial cancer, but no significant association was observed with respect to all-cause cancer as well as other types of cancers. (2) Moreover, nitrogen-containing bisphosphonates could reduce the risk of breast cancer by 6% and endometrial cancer by 30%; non-nitrogen-containing bisphosphonates could also reduce the risk of breast cancer by 12%. However, non-nitrogen-containing bisphosphonates might be associated with an increased risk of liver and pancreas cancer. (3) The use of bisphosphonates upto at least 1 year had a greater protective effect on breast cancer than their use for less than 1 year, but such result was not found in other cancers.

### Consistent and inconsistent with current studies

With regard to all-cause cancer, our results are largely consistent with previous meta-analyses in similar contexts, supporting that the association between bisphosphonates and the risk of all-cause cancer is not statistically significant.^26^ The difference is that their study only included 13 cohort studies. Because this study had more sample size and the total number of cancer cases, we could examine more types of cancers and obtain higher statistical power. As we found that the sample size might be one of the sources of heterogeneity, we speculate that the use of bisphosphonates has a weak protective effect on all-cause cancer, which needs more sample size to explore this association.

With regard to female cancers, our findings are consistent with previous studies that bisphosphonates could reduce the risk of breast^23,26,67,68^ and endometrial cancer,^22,24^ but no significant link was observed in ovarian cancer.^24^ This might be due to the oestrogen receptor (ER) involved in the anticancer effect of bisphosphonates.^69^ Furthermore, the study by Chlebowski et al.^41^ showed that bisphosphonates reduced the risk of ER-positive breast cancer by 30% (HR = 0.70, 95% CI: 0.52–0.95, *P* = 0.02). However, the data on the association between bisphosphonates and ER-positive female cancers are poor, as we could not conduct subgroup analysis based on ER. Further studies are needed to focus on the use of bisphosphonates and their protective effect on the different subtypes of female cancers to confirm these findings.

The effect of bisphosphonates on gastrointestinal cancers is controversial. The study by Oh et al.^25^ showed that there was no significant association between bisphosphonates and the risk of oesophageal cancer (RR = 0.96, 95% CI: 0.65–1.42), which was consistent with our results and other three meta-analyses.^20,21,26^ However, Andrici et al.^70^ found that bisphosphonates might increase the risk of oesophageal cancer (OR = 1.74, 95% CI: 1.19–2.55). Similarly, the results regarding the association between bisphosphonates and the risk of gastric cancer are inconsistent.^12,54,55^ Clinical reports have found that bisphosphonates can cause gastrointestinal problems, such as erosive oesophagitis and gastric ulcers;^71,72^ these patients are more likely to receive the upper gastrointestinal endoscopy to accelerate the discovery of upper gastrointestinal cancer. In addition, because oesophageal adenocarcinoma of the distal oesophagus is very similar to the adenocarcinoma at the junction of the gastro-oesophagus, it is difficult to accurately distinguish them in clinical diagnosis. Original studies rarely report the results of subgroup analysis of the precise site of upper gastrointestinal cancer, as we are unable to perform pool analysis. We should focus on distinguishing the particular subtypes of oesophageal and gastric cancer when analysing the effects of bisphosphonates on gastric and oesophageal cancer in future studies. With respect to colorectal cancer, our study indicated that bisphosphonates could reduce the risk of colorectal cancer by 11%, which was similar with other five meta-analyses,^15–19^ but Deng et al.^26^ and Oh et al.^25^ suggested that the association between bisphosphonates and the risk of colorectal cancer was not statistically significant. It is well known that bisphosphonates are commonly used for osteoporosis because they have lower bone mineral density (BMD). Previous studies have shown that BMD is associated with cancer, which means that if not adjusted, it might be a confounding factor that masks the protective effect of bisphosphonates.^73,74^ For other types of cancers, the results are basically similar to previous studies, and the number of related studies is few.^26,75^

### Types and duration of bisphosphonates and confounders

Experimental studies have suggested that nitrogen-containing bisphosphonates have potential antitumour effects. They could reduce the viability of tumour cells by binding to the kinase domain of the human epidermal growth factor receptor 1/2 (HER1/2), and resulting in an overall reduction in global downstream signalling that was driven by overexpression of the HER family,^76,77^ such as lung,^78^ breast^79^ and colorectal cancer.^80^ We only observed that nitrogen-containing bisphosphonates could reduce 30% risk of endometrial cancer and 6% risk of breast cancer. However, there was no significant association between the use of nitrogen-containing bisphosphonates and risk of colorectal and lung cancer in our study. Simultaneously, we found that non-nitrogen-containing bisphosphonates might be associated with an increased risk of liver and pancreas cancer, which might be due to the greater toxicity on liver and pancreas. Clodronate (non-nitrogen-containing bisphosphonate) might cause evaluation of aminotransferase.^81^ Moreover, there were rare studies of liver disease developing in patients who use non-nitrogen-containing bisphosphonate.^82,83^ In addition, from the limited information, we could not find any possible mechanism to explain the association between non-nitrogen-containing bisphosphonates and the risk of pancreas cancer, but we should pay attention to the safety of using this drug. In the future, it might be necessary to further explore the pharmacological mechanisms of non-nitrogen-containing bisphosphonates.

Notably, we did not find that the duration of bisphosphonates had an effect on specific cancers, except breast cancer. For breast cancer, our results were consistent with a meta-analysis by Liu et al.,^68^ which showed that using bisphosphonates upto at least 1 year (RR = 0.75, 95% CI: 0.66–0.84) seemed to be greater protective on breast cancer than using them for less than 1 year (RR = 0.90, 95% CI: 0.84–0.97). However, Ou et al.^23^ suggested that bisphosphonate was not associated with risk of breast cancer when the usage time was less than 1 year (RR = 0.93, 95% : 0.86–1.00), but a significant 26% reduction was found upto at least 1 year (RR = 0.74, 95% CI: 0.66–0.83), which was consistent with the study by Newcomb et al.^61^ Patients with the long-term use of bisphosphonates might have a healthier lifestyle and higher adherence to the drugs, so the benefits observed in the analysis might be overestimated.^84^ Thus, further studies are needed to consider this potential bias and find the best duration and dose of bisphosphonates.

In this study, the evidence we have on the use of bisphosphonates and risk of cancers is based mainly on observational studies. Many studies are not able to adequately control confounding factors related to cancers. Wright et al.^60^ demonstrated a small but significantly increased risk of oesophageal cancer in women, not in men. Generally, women prescribe bisphosphonates for prevention and treatment of osteoporosis, while men are more likely to prescribe bisphosphonates for iatrogenic osteoporosis.^85,86^ Hence, most of the participants in included studies were women, and the number of men was much smaller. To date, there is still a lack of studies on the association between bisphosphonates and risk of cancers in people of different genders. Secondly, some studies have reported that supplemental calcium and vitamin D might have a protective effect on colorectal cancer, which are usually prescribed with bisphosphonates.^87,88^ Some included studies did not control for calcium and vitamin D when analysing the association between bisphosphonates and the risk of colorectal cancer.^52,54,58^ In addition, only a few studies adjust the use of statins, hormone replacement therapy (HRT) and family history of cancers, which are also related to cancers.^44–46,49^

### Strengths and limitations

This study has several strengths. First, there are many articles and a large number of participants included in this study, which increases the statistical power of the analysis. Furthermore, we explore the sources of heterogeneity by meta-regression and subgroup analysis. Finally, this systematic review and meta-analysis has an update to the summary articles in this field. However, several limitations of this study must be mentioned. Firstly, our study includes observational studies (cohort studies, case–control studies, nested case–control studies and case cohort studies) that might differ in the design of researches and lack individual information. In addition, evidence from observational studies is weaker than that from randomised controlled trials. Secondly, the meta-analysis of observational studies is susceptible to confounding factors that exist in original studies because most of the included studies use large and anonymous databases.^89^ Although all included studies attempt to adjust confounding factors, there are still potential confounders that are not considered, which might affect our results. Thirdly, we are unable to perform subgroup analysis of precise sites of oesophageal and gastric cancer, because the original studies rarely report the results in this aspect. Finally, although we have explored several sources of heterogeneity with meta-regression model and subgroup analysis, it still cannot fully explain the heterogeneity in the studies.

## Conclusions

In conclusion, the results of this meta-analysis suggest that the use of bisphosphonates is associated with a decreased risk of colorectal, breast and endometrial cancer, but not significantly associated with the risk of oesophageal and other types of cancers. Furthermore, we find that nitrogen-containing bisphosphonates appear to have more antitumour effects, but non-nitrogen- containing bisphosphonates might be associated with an increased risk of liver and pancreas cancer. In addition, the use of bisphosphonates for at least 1 year has a greater protective effect on breast cancer than their use for less than 1 year. We recommend that further large prospective cohort studies are needed to explore the causal association between bisphosphonates and cancers, and more studies of potential mechanism are required. After a careful benefit-and-risk assessment, whether nitrogen-containing bisphosphonates are expected to be used in populations at a high risk for the malignancies needs further discussion.

## Supplementary information

Supplementary files

## Data Availability

The datasets used and analysed during the current study are available from the corresponding author on reasonable request.
