# Supplementary material for: Bisphosphonates and risk of cancers: a systematic review and meta-analysis
Source: Br J Cancer. 2020 Sep 9;123(10):1570–81. doi: 10.1038/s41416-020-01043-9 (PMC7652831; doi:10.1038/s41416-020-01043-9)
Supplement: Supplementary file 1 — Supplementary files [file 41416_2020_1043_MOESM1_ESM.docx]

**Supplementary Table S1.** Search terms of the current systematic review and meta-analysis

| Exposure | Outcomes |
| --- | --- |
| Diphosphonates | Neoplasms |
| Diphosphonate | Neoplasm |
| Bisphosphonate | Malignant neoplasms |
| Bisphosphonates | Malignant neoplasm |
| Bisphosphonic acid derivative | Neoplasia |
| Bisphosphonate derivative | Malignant neoplasia |
| Diphosphonate derivative | Malignant neoplastic disease |
| Alendronate | Malignant tumor |
| Clodronate | Malignant tumors |
| Etidronate | Malignant tumour |
| Ibandronate | Malignant tumours |
| Incadronate | Tumor |
| Lidadronate | Tumors |
| Medronate | Tumour |
| Minodronate | Tumours |
| Neridronate | Cancer |
| Olpadronate | Cancers |
| Pamidronate | Carcinomas |
| Risedronate | Carcinoma |
| Tiludronate | Malignancies |
| Zoledronate | Malignancy |
| Technetium Tc 99m Medronate | NA |

*NA* not available.

**Supplementary Table S2.** Adjusted confounding factors of the 34 included studies

| Study [year] | Maximum adjusted confounding factors |
| --- | --- |
| Fortuny et al. [2009]^63^ | Age, BMI, education, race, menarche, hormone therapy use, oral contraceptives use, age at menopause, parity, smoking, family history of endometrial cancer |
| Newcomb et al. [2010]^61^ | Age, parity, BMI, family history of breast cancer, menopausal status, age at menopause, type of hormone use, mammography, osteoporosis, smoking history, height change |
| Rennert et al. [2010]^65^ | Age, residence, ethnic, BMI, age at first pregnancy, family history, sports activity, fruits |
| Rennert et al. [2011]^64^ | Age, sex, residence, ethnic, family history, BMI, sports participation, vegetable consumption, aspirin, statin, vitamin D, postmenopausal hormones |
| Wright et al. [2012]^60^ | Age, sex, BMI, smoking, alcohol intake, PPI, H. pylori status, dyspepsia |
| Rennert et al. [2014]^62^ | Age, residence, ethnic group, BMI, HRT, breast feeding duration, number of pregnancies |
| Green et al. [2010]^59^ | Age, sex, general practice, observation period, smoking, alcohol, BMI |
| Nguyen et al. [2010]^56^ | Age, the BE index date, race, non-cancer disease comorbidity index, PPI, NSAIDs |
| Chen et al. [2011]^50^ | Age, sex, cohort entry year |
| Singh et al. [2012]^52^ | Age, sex, length of stay in the province, the number of ambulatory care physician visits, NSAIDs, Charlson comorbidity index, history of lower gastrointestinal endoscopy |
| Vinogradova et al. [2013]^53^ | Age, sex, practice, calendar year, BMI, smoking, alcohol, ethnicity, rheumatoid arthritis, osteoporosis and fractures, use of other osteoporosis drugs, vitamin D, NSAIDs, corticosteroids, acid-lowering drugs, years of data, family history |

**Supplementary Table S2.** (continued)

| Study [year] | Maximum adjusted confounding factors |
| --- | --- |
| Vinogradova et al. [2013]^54^ | Age, sex, practice, calendar year, smoking, alcohol, osteoporosis and drugs associated with it, history of upper gastrointestinal disorders, and family history of relevant cancer |
| Vogtmann et al. [2015]^55^ | Age, gender, duration of membership prior to index date, race, region of residence, smoking, alcohol, Charlson comorbidity index, PPI, NSAIDs, H2-Receptor antagonists, history of gastroesophageal reflux disease or osteoporosis, previous upper endoscopy |
| Jung et al. [2016]^12^ | Age, sex, income level by the type of insurance, diabetes mellitus without complications, diabetes mellitus with complications, rheumatoid arthritis, and Paget disease of bone |
| Busby et al. [2017]^57^ | Age, gender, year of diagnosis, general practice, statin, aspirin, the presence of myocardial infarction, heart failure, peripheral vascular disease, cerebrovascular disease, connective tissue disease, dementia, chronic obstructive pulmonary disease, rheumatoid arthritis, diabetes, renal disease, liver disease |
| Vogtmann et al. [2017]^58^ | Age, membership duration, race, residential region, BMI, smoking, alcohol, Charlson comorbidity index, NSAIDs, previous lower endoscopy |
| Chung et al. [2018]^51^ | Age, sex, comorbidity, history of NSAIDs or aspirin |
| Chiang et al. [2012]^66^ | Age, sex, comorbidities, the enrollment date, hypertension, diabetes, chronic obstructive pulmonary disease, estrogen, dyslipidemia, chronic kidney disease, coronary artery disease, colorectal polyp, benign breast disease, morbid obesity, statin |
| Abrahamsen et al. [2009]^40^ | Age, sex, fracture type, Charlson comorbidity index, the number of concomitant medications, the time to an event, death, or the end of the study |
| Cardwell et al. [2010]^38^ | Age, sex, general practice, BMI, alcohol, smoking, hormone therapy, NSAIDs, BE diagnosis, gastroesophageal reflux disease diagnosis, H2 receptor antagonist prescription, PPI |
| Chlebowski et al. [2010]^41^ | Age, ethnicity, smoking, alcohol, physical activity, BMI, mammogram, prior estrogen alone use, prior estrogen-progestin use, calcium, vitamin D, 5-year risk of hip fracture, Gail 5-year risk of breast cancer |
| Vestergaard et al. [2011]^11^ | Age, sex, alcoholism, smoking, antacid drugs, aspirin, NSAIDs, occupation, marital status, income, gastric surgery history |
| Vestergaard et al. [2011]^48^ | Age, gender, alcoholism, hormone therapy, irradiation, chemotherapy |

**Supplementary Table S2.** (continued)

| Study [year] | Maximum adjusted confounding factors |
| --- | --- |
| Abrahamsen et al. [2012]^37^ | Age, sex, comedications, Charlson comorbidity index, PPI, history of upper endoscopy |
| Cardwell et al. [2012]^13^ | Age, sex, general practice, BMI, alcohol, smoking, NSAIDs, glucocorticoid steroid, vitamin D prescription, calcium |
| Khalili et al. [2012]^45^ | Age, race, BMI, smoking, alcohol, physical activity, aspirin, statin, calcium, vitamin D, folate, red meat, family history of colon cancer, history of osteoporosis, HRT, history of polyps, history of screening |
| Lee et al. [2012]^39^ | Age, gender |
| Pazianas et al. [2012]^47^ | Age, sex, year of beginning treatment, Charlson index, known ulcerative colitis, known Crohn’s disease, known coeliac disease, hormone replacement therapy, amount of prednisolone, NSAIDs, aspirin |
| Passarelli et al. [2013]^14^ | Age, race, education, BMI, smoking, alcohol, physical activity, NSAIDs, aspirin, family history of colorectal cancer, estrogen‐only use, estrogen‐progestin use, history of endoscopy, history of mammography, calcium, vitamin D, 5‐year hip fracture probability |
| Alford et al. [2015]^46^ | Age, race, BMI, HRT, smoking |
| Newcomb et al. [2015]^43^ | Age, BMI, race, education, smoking, estrogen-only use, estrogen-progestin use, oral contraceptive use, parity, and mammography, all measured at baseline, 5-year hip fracture probability |
| Fournier et al. [2017]^49^ | Age, years of schooling, alcohol, BMI, history of fractures, osteoporosis and bone densitometries, parity, use of oral contraceptives, time since menopause, history of breast cancer in first-degree relatives, history of benign breast disease, HRT, mammogram, raloxifene, vitamin D |
| Tao et al. [2018]^42^ | Age, ethnicity, education, BMI, smoking, number of cigarettes per day, duration of regular smoking in years, alcohol, physical activity, calcium, vitamin D, statin, hormone treatment status |
| Bae et al. [2019]^44^ | Age, BMI, smoking, alcohol, exercise frequency, blood pressure, cholesterol, diabetes, quartiles of socioeconomic status, Charlson comorbidity index, hormonal replacement therapy, oral steroid, aspirin, NSAIDs, statin, calcium, vitamin D |

*NSAIDs* nonsteroidal anti-inflammatory drugs; *BMI* body mass index; *PPI* proton pump inhibitor; *HRT* hormone replacement therapy; *BE* Barrett’s esophagus.

**Supplementary Table S3.** Quality assessment of 6 case-control studies by Newcastle-Ottawa Scale (NOS)

| Study | Selection | | | | Comparability ^a^ | Exposure | | | Total |
| --- | --- | --- | --- | --- | --- | --- | --- | --- | --- |
|  | Case definition adequate | Representativeness of cases | Selection of controls | Definition of controls |  | Ascertainment of exposure | Same method ^b^ | Non-response rate |  |
| Fortuny et al. [2009]^63^ | √ | √ |  | √ | √ √ | √ | √ | √ | 8 |
| Newcomb et al. [2010]^61^ | √ | √ |  | √ | √ √ | √ | √ |  | 7 |
| Rennert et al. [2010]^65^ | √ | √ | √ | √ | √ | √ | √ |  | 7 |
| Rennert et al. [2011]^64^ | √ | √ | √ | √ | √ √ | √ | √ |  | 8 |
| Wright et al. [2012]^60^ | √ | √ | √ | √ | √ | √ | √ | √ | 8 |
| Rennert et al. [2014]^62^ | √ | √ | √ | √ | √ |  | √ |  | 6 |

^a^ √ study controls for age and gender; √√study controls for age, gender and other factors.

^b^ Same method of ascertainment for cases and controls.

**Supplementary Table S4.** Quality assessment of 1 case cohort study, 11 nested case-control studies and 16 cohort studies by Newcastle-Ottawa Scale (NOS)

| Study | Selection | | | | Comparability ^a^ | Exposure | | | Total |
| --- | --- | --- | --- | --- | --- | --- | --- | --- | --- |
|  | Exposed cohort | Non-exposed cohort | Ascertainment of exposure | Outcome of interest |  | Assessment of outcome | Follow-up long enough | Adequacy of follow up |  |
| Nested case-control studies |  |  |  |  |  |  |  |  |  |
| Green et al. [2010]^59^ | √ | √ | √ | √ | √ | √ | √ |  | 7 |
| Nguyen et al. [2010]^56^ |  | √ | √ | √ | √ √ | √ | √ | √ | 8 |
| Chen et al. [2011]^50^ | √ | √ | √ |  | √ |  | √ | √ | 6 |
| Singh et al. [2012]^52^ | √ | √ | √ | √ | √ | √ | √ | √ | 8 |
| Vinogradova et al. [2013]^53^ | √ | √ | √ | √ | √ √ | √ | √ |  | 8 |
| Vinogradova et al. [2013]^54^ | √ | √ | √ | √ | √ √ | √ | √ |  | 8 |
| Vogtmann et al. [2015]^55^ | √ | √ | √ | √ | √ √ | √ | √ |  | 8 |
| Jung et al. [2016]^12^ | √ | √ | √ | √ | √ | √ | √ | √ | 8 |
| Busby et al. [2017]^57^ | √ | √ | √ | √ | √ √ | √ | √ |  | 8 |
| Vogtmann et al. [2017]^58^ | √ | √ | √ | √ | √ √ | √ | √ |  | 8 |
| Chung et al. [2018]^51^ | √ | √ | √ | √ | √ √ | √ | √ | √ | 9 |
| Case cohort study |  |  |  |  |  |  |  |  |  |
| Chiang et al. [2012]^66^ | √ | √ | √ | √ | √ | √ | √ | √ | 8 |

**Supplementary Table S4.** (continued)

| Study | Selection | | | | Comparability ^a^ | Exposure | | | Total |
| --- | --- | --- | --- | --- | --- | --- | --- | --- | --- |
|  | Exposed cohort | Non-exposed cohort | Ascertainment of exposure | Outcome of interest |  | Assessment of outcome | Follow-up long enough | Adequacy of follow up |  |
| Cohort studies |  |  |  |  |  |  |  |  |  |
| Abrahamsen et al. [2009]^40^ | √ | √ | √ |  | √ | √ | √ |  | 6 |
| Cardwell et al. [2010]^38^ | √ | √ | √ | √ | √ √ | √ | √ |  | 8 |
| Chlebowski et al. [2010]^41^ | √ | √ | √ | √ | √ √ | √ | √ |  | 8 |
| Vestergaard et al. [2011]^11^ | √ | √ | √ | √ | √ √ | √ | √ | √ | 9 |
| Vestergaard et al. [2011]^48^ | √ | √ | √ | √ | √ √ | √ | √ | √ | 9 |
| Abrahamsen et al. [2012]^37^ | √ | √ | √ | √ | √ | √ | √ | √ | 8 |
| Cardwell et al. [2012]^13^ | √ | √ | √ | √ | √ √ | √ | √ |  | 8 |
| Khalili et al. [2012]^45^ |  | √ |  | √ | √ √ | √ | √ | √ | 7 |
| Lee et al. [2012]^39^ | √ | √ |  | √ | √ | √ | √ | √ | 7 |
| Pazianas et al. [2012]^47^ | √ | √ | √ | √ | √ √ | √ | √ | √ | 9 |
| Passarelli et al. [2013]^14^ | √ | √ | √ | √ | √ √ | √ | √ |  | 8 |
| Alford et al. [2015]^46^ | √ | √ |  | √ | √ | √ | √ |  | 6 |
| Newcomb et al. [2015]^43^ | √ | √ | √ | √ | √ √ | √ | √ |  | 8 |

**Supplementary Table S4.** (continued)

| Study | Selection | | | | Comparability ^a^ | Exposure | | | Total |
| --- | --- | --- | --- | --- | --- | --- | --- | --- | --- |
|  | Exposed cohort | Non-exposed cohort | Ascertainment of exposure | Outcome of interest |  | Assessment of outcome | Follow-up long enough | Adequacy of follow up |  |
| Fournier et al. [2017]^49^ | √ | √ | √ | √ | √ √ |  | √ |  | 7 |
| Tao et al. [2018]^42^ | √ | √ | √ | √ | √ √ | √ | √ |  | 8 |
| Bae et al. [2019]^44^ | √ | √ | √ | √ | √ √ | √ | √ | √ | 9 |

^a^ √ Study controls for age and gender; √√Study controls for age, gender and other factors.


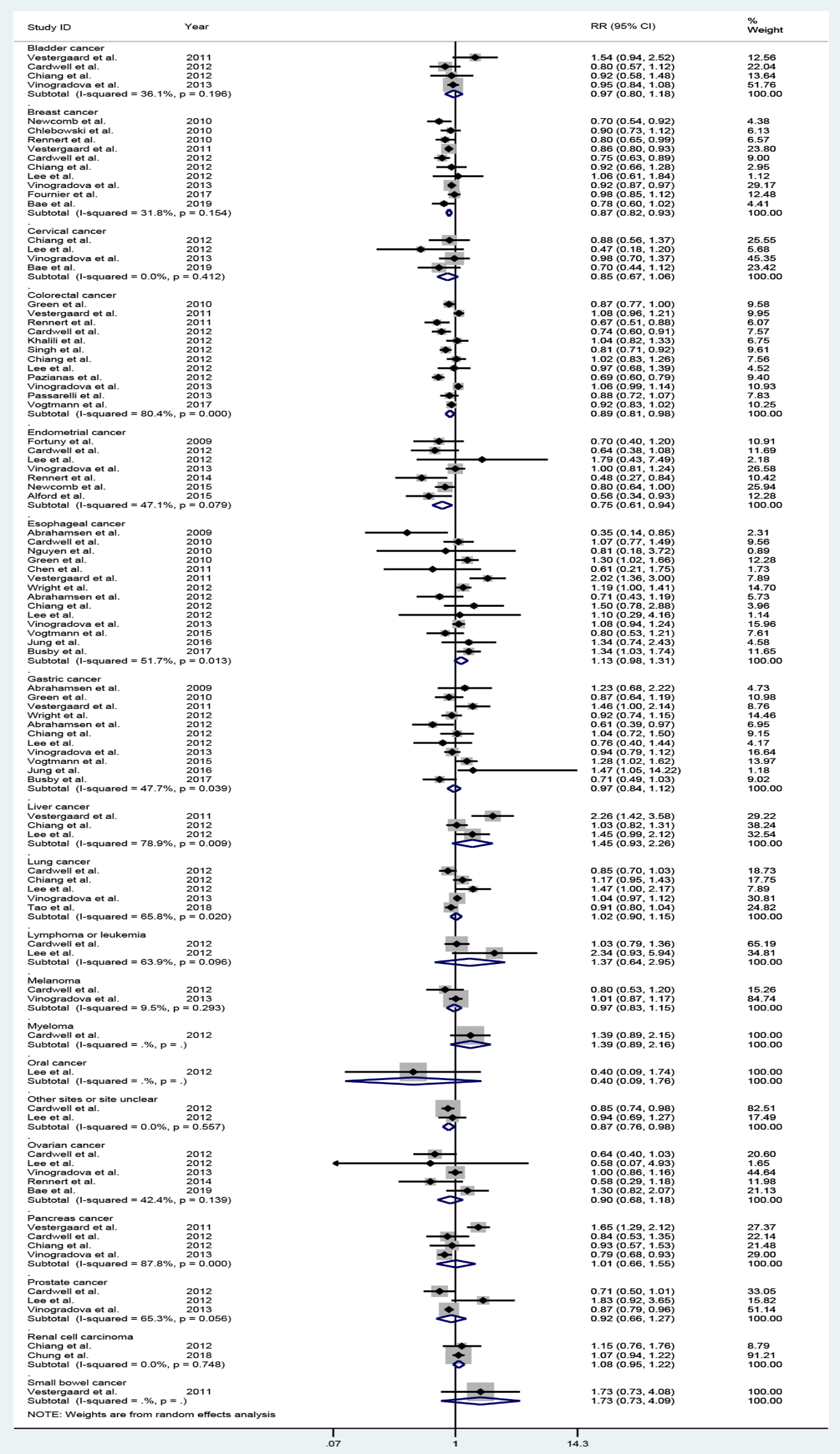


**Supplementary Fig. S1** **Forest plot of summary risk estimates in overall cancers.** *RR* risk ratio; *CI* confidence interval. The forest plot shows the RRs of studies comparing individuals using bisphosphonates to those without bisphosphonates. The publication year of studies are shown in the figure. Each small rhombuses represents the RR for each studies, with the location of the rhombuses representing both the direction and magnitude of the effect size and the horizontal line representing their 95% CIs. The square represents the weight of studies, and the hollow rhombus represents the pooled RRs. The maximally adjusted risk estimates with 95% CIs were pooled by using random-effect models to obtain a more conservative outcome.

**Supplementary Fig. S2** **Sensitivity analysis of the association between the use of bisphosphonates and risk of breast cancer.** The graph of sensitivity analysis shows results performed by omitting one study and calculating the risk estimates with 95% confidence intervals of the remaining studies. The dot represents the risk estimates of remaining studies with omitting one study, and the dotted line represents their 95% confidence intervals. The vertical line represents the pooled risk estimates with 95% confidence interval.

**Supplementary Fig. S3** **Sensitivity analysis of the association between the use of bisphosphonates and risk of colorectal cancer.** The graph of sensitivity analysis shows results performed by omitting one study and calculating the risk estimates with 95% confidence intervals of the remaining studies. The dot represents the risk estimates of remaining studies with omitting one study, and the dotted line represents their 95% confidence intervals. The vertical line represents the pooled risk estimates with 95% confidence interval.

**Supplementary Fig. S4** **Sensitivity analysis of the association between the use of bisphosphonates and risk of esophageal cancer.** The graph of sensitivity analysis shows results performed by omitting one study and calculating the risk estimates with 95% confidence intervals of the remaining studies. The dot represents the risk estimates of remaining studies with omitting one study, and the dotted line represents their 95% confidence intervals. The vertical line represents the pooled risk estimates with 95% confidence interval.

**Supplementary Fig. S5** **Sensitivity analysis of the association between the use of bisphosphonates and risk of gastric cancer.** The graph of sensitivity analysis shows results performed by omitting one study and calculating the risk estimates with 95% confidence intervals of the remaining studies. The dot represents the risk estimates of remaining studies with omitting one study, and the dotted line represents their 95% confidence intervals. The vertical line represents the pooled risk estimates with 95% confidence interval.
